# Intrapancreatic Ganglia and Neural Regulation of Pancreatic Endocrine Secretion

**DOI:** 10.3389/fnins.2019.00021

**Published:** 2019-02-20

**Authors:** Wenjing Li, Guangjiao Yu, Yudan Liu, Lei Sha

**Affiliations:** ^1^School of Pharmacy, China Medical University, Shenyang, China; ^2^China Medical University-The Queen’s University of Belfast Joint College, China Medical University, Shenyang, China

**Keywords:** intrapancreatic ganglia, extrapancreatic nerves, pancreas, islet, insulin

## Abstract

Extrapancreatic nerves project to pancreatic islets directly or converge onto intrapancreatic ganglia. Intrapancreatic ganglia constitute a complex information-processing center that contains various neurotransmitters and forms an endogenous neural network. Both intrapancreatic ganglia and extrapancreatic nerves have an important influence on pancreatic endocrine function. This review introduces the histomorphology, innervation, neurochemistry, and electrophysiological properties of intrapancreatic ganglia/neurons, and summarizes the modulatory effects of intrapancreatic ganglia and extrapancreatic nerves on endocrine function.

## Introduction

Intrapancreatic ganglia were first described by Langerhans in 1869 and have been studied over several decades (Sha et al., 1995, 1996, 1997, 2001b; Liu and Kirchgessner, 1997; Love and Szebeni, 1999b; Sha and Szurszewski, 1999; Yi et al., 2005; Shen et al., 2016). While intrapancreatic ganglia have been traditionally considered to be simple parasympathetic relays, growing evidence suggests that, similar to the enteric nervous system (ENS), intrapancreatic ganglia may constitute a complex information-processing center containing various neurotransmitters and form an endogenous neural network (Gylfe and Tengholm, 2014; Shen et al., 2016; Tang et al., 2018b). Although the influence of extrapancreatic nerves, especially autonomic nerves, on pancreatic endocrine function has been extensively studied, the role of intrapancreatic ganglia in endocrine and exocrine functions has garnered far less attention (Sha et al., 2001b; Love et al., 2007; Gylfe and Tengholm, 2014; Rodriguez-Diaz and Caicedo, 2014). In this review, we will focus on research studies from the past 30 years. We will introduce the histomorphology, innervation, neurochemistry, and electrophysiological properties of intrapancreatic ganglia/neurons, and summarize the modulatory effects of intrapancreatic ganglia and extrapancreatic nerves on endocrine function.

## Anatomy and Morphology of Intrapancreatic Ganglia

Intrapancreatic ganglia are composed of pancreatic neurons, glial cells, and extrinsic and intrinsic nerve fibers. They are located over or alongside nerve trunks in the interlobular, acinar, or insular connective tissues, or within lobules and islets (Liu and Kirchgessner, 1997; Tang et al., 2018a). Intrapancreatic ganglia can be oval, round, or polygonal in shape, and vary in size (Shen et al., 2016). Most intrapancreatic ganglia are found alongside or inside nerve trunks that are branches of larger nerve trunks traveling along the pancreaticoduodenal artery in the head of the pancreas (Liu and Kirchgessner, 1997). Ganglia are also found along nerve bundles from the celiac and superior mesenteric plexuses, and the splenic nerves in the body and tail of the pancreas (Love et al., 2007). The ganglia in the head are larger and contain more neurons than those in the body and tail of the pancreas (Sha et al., 1996). In the rabbit pancreas, the larger ganglia (≥6 neurons) often appear to be encapsulated and connect to larger nerve trunks, while the smaller ganglia are similar to grape clusters; single pancreatic neurons are also found within islets (Love and Szebeni, 1999b). The peri-lobular ganglia are larger than intra-parenchymal ganglia. The ganglia of patients with type 2 diabetes are larger than those of individuals without diabetes (Tang et al., 2018a). The number of neurons in each pancreatic ganglion is different in different species. For example, rabbit ganglia contain 1 to 35 neurons per ganglion [mean ± standard error of the mean (SEM), 4 ± 1] (Love and Szebeni, 1999b), while rat ganglia contain 2–24 neurons per ganglion (mean ± SEM, 8.8 ± 0.5) (Shen et al., 2016). Although the neurons are typically oval or round, triangular neurons are also found. Most of the neurons are bipolar or multipolar, and unipolar neurons are rare. While the dendritic neuropil of neurons is variable in shape, most of the dendrites are typically short and stubby (Sha et al., 1996). Most nerve fibers of intrapancreatic ganglia are unmyelinated. The unmyelinated nerve bundles have a fine axonal structure, with a large number of microtubules present in the center. Some axons contain both small, clear vesicles and large dense-cored vesicles, while others appear to contain only small, clear vesicles (Love et al., 2007). The axons of pancreatic neurons project to all pancreatic effector cells, including islets, pancreatic ducts, acini, blood vessels, and other intrapancreatic ganglia, indicating that intrapancreatic ganglia may play an important role in pancreatic functions, including endocrine function (Niebergall-Roth and Singer, 2001; Love et al., 2007).

## Innervation of Intrapancreatic Ganglia

### Sympathetic Efferent Fibers

**Figure 1:**
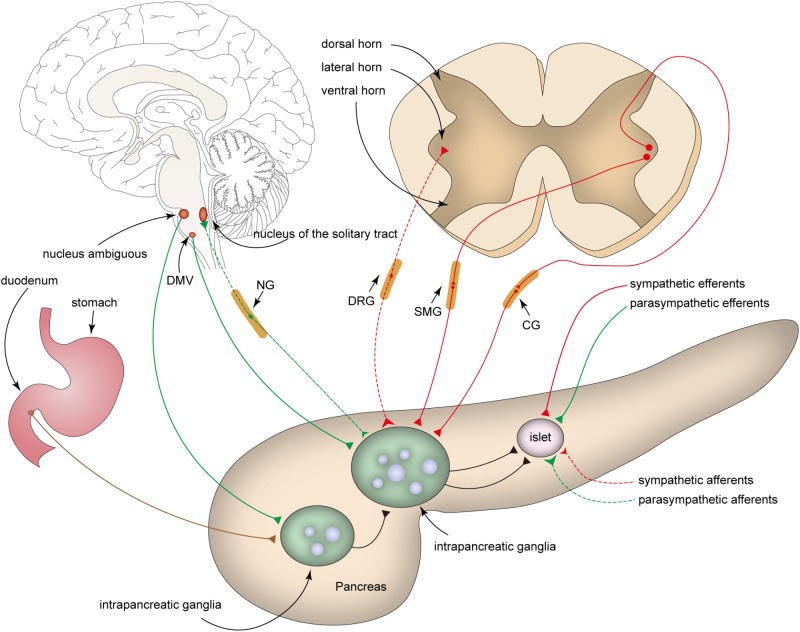
Innervation of intrapancreatic ganglia. Intrapancreatic ganglia receive inputs from sympathetic fibers (red), parasympathetic fibers (green), enteric plexus (brown), and nerves of other intrapancreatic ganglia. The parasympathetic efferent fibers (green solid line) originate from the dorsal motor nucleus of the vagus (DMV) and ambiguous nucleus, whereas the cell bodies of sympathetic efferent fibers (red solid line) project from the lateral horn of the spinal cord to the celiac ganglia (CG), superior mesenteric ganglia (SMG), or paravertebral ganglia (not show in the Figure). The cell bodies of parasympathetic (green dashed line) and sympathetic (red dashed line) afferents are positioned in the nodose ganglia (NG) and the dorsal root ganglia (DRG), respectively.

Preganglionic efferent sympathetic fibers project from cell bodies in the lateral horn of the spinal cord (C_8_-L_3_) to paravertebral and prevertebral sympathetic ganglia (Llewellyn-Smith, 2009; Figure 1). The fibers projecting from the prevertebral ganglia (the celiac and superior mesenteric ganglia) enter the pancreas directly or with other autonomic nerves (Ahrén, 2000; Gilon and Henquin, 2001; Love et al., 2007). The celiac plexus mainly includes four branches: the anterior hepatic plexus, posterior hepatic plexus, splenic plexus, and plexus accompanying the transverse pancreatic artery (Dolensek et al., 2015). These branches are distributed in different locations of the human pancreas. The head of the pancreas receives the anterior and posterior hepatic plexuses, which are distributed along the hepatic artery and located in the dorsal aspect of the portal vein. The body and tail of the pancreas are innervated by the other two plexuses. Some nerves originate from the superior mesenteric ganglia and enter the uncinate process of the pancreas along the inferior pancreaticoduodenal artery. There also exist plexuses that surround the main pancreatic duct, but pass through the pancreas independently of the blood vessels (Yi et al., 2003). The postganglionic sympathetic fibers project to intrapancreatic ganglia, islets, ducts, and blood vessels, and release several neurotransmitters such as norepinephrine (NE), galanin, and neuropeptide Y (NPY) (Ahrén, 1999; Di Cairano et al., 2016). Preganglionic sympathetic fibers also enter the pancreas directly and terminate at the intrapancreatic ganglia (Havel et al., 1989).

### Parasympathetic Efferent Fibers

Most preganglionic parasympathetic fibers supplying the pancreas originate from the dorsal motor nucleus of the vagus (DMV) in the medulla, and their cell bodies are rarely observed in the nucleus ambiguous (Love et al., 2007; Chandra and Liddle, 2013; Rodriguez-Diaz and Caicedo, 2014; Ma and Vella, 2018; Figure 1). Vagus nerve fibers pass through the hiatus of the esophagus into the abdomen and are divided into five distinct branches that subsequently innervate different organs. The vagus nerve fibers that innervate the pancreas include the hepatic and bilateral gastric branches (primary), and bilateral celiac branches (secondary) (Niebergall-Roth and Singer, 2001; Love et al., 2007; Chandra and Liddle, 2014).

A number of parasympathetic preganglionic fibers terminate at the intrapancreatic ganglia to form synapses (Chandra and Liddle, 2009). Acetylcholine (ACh) is released from both the preganglionic and postganglionic parasympathetic nerve terminals. In addition, vasoactive intestinal polypeptide (VIP), gastrin-releasing peptide (GRP), pituitary adenylate cyclase-activating polypeptide (PACAP), and nitric oxide (NO) are released from the postganglionic parasympathetic nerve fibers (Wang et al., 1999; Gilon and Henquin, 2001; Love et al., 2007; Di Cairano et al., 2016). NPY and galanin exist in both sympathetic and parasympathetic nerve fibers of the pancreas in pigs and mice (Pettersson et al., 1987; Sheikh et al., 1988; Ahrén, 2000).

### Enteropancreatic Plexus

The ENS within the wall of the gastrointestinal tract is often considered to be the “local brain” that regulates gastrointestinal functions. Enteric neurons in the myenteric plexus of the gastric antrum and proximal duodenum also project out of the gut to the intrapancreatic ganglia (Kirchgessner and Gershon, 1990; Chandrasekharan and Srinivasan, 2007). Cholinergic enteropancreatic neurons form excitatory nicotinic synapses on pancreatic neurons. In addition, some enteropancreatic nerves, such as the PACAP-containing nerves, are peptidergic. PACAP is often considered as a neuromodulator that strengthens pancreatic secretion (Kirchgessner and Liu, 2001). The fibers of serotonergic enteropancreatic neurons form inhibitory axo-axonic synapses to inhibit amylase secretion (Kirchgessner and Gershon, 1995). Enteropancreatic neurons form nicotinic synapses in intrapancreatic ganglia; however, other excitatory pathways remain unknown. The role of enteric nerves in controlling the endocrine functions of the pancreas has not been completely elucidated. The ENS may be a therapeutic target for the regulation of glucose metabolism (Abot et al., 2018a,b).

### Afferent Nerve Fibers

Intrapancreatic ganglia receive both sympathetic and parasympathetic afferents, most of which are sensitive to capsaicin. These afferent fibers transmit sensory information from the pancreas to the central nervous system (Dolensek et al., 2015).

Sympathetic afferents are thought to exit the pancreas along the postganglionic sympathetic fibers within the splanchnic nerves and the celiac plexus to the dorsal root ganglia (DRG, T_6_-L_2_) (Love et al., 2007; Rodriguez-Diaz and Caicedo, 2014; Dolensek et al., 2015). Pancreatic parasympathetic afferents originate from the nodose ganglia (NG) (Lartigue, 2016). While pancreatic parasympathetic afferent fibers primarily supply the blood vessels, ducts, acini, and islets, few parasympathetic afferents are found in intrapancreatic ganglia (Love et al., 2007). Although the central targets of vagal afferents remain largely unclear, the nucleus of the solitary tract (NTS) is considered to be the main relay center for vagal afferent nerves (Renehan et al., 1995; Dolensek et al., 2015). Sympathetic and parasympathetic afferent fibers contain either substance P (SP) or calcitonin gene-related peptide (CGRP), or both (Rodriguez-Diaz and Caicedo, 2014).

## Neurotransmitters of Intrapancreatic Ganglia

**Figure 2:**
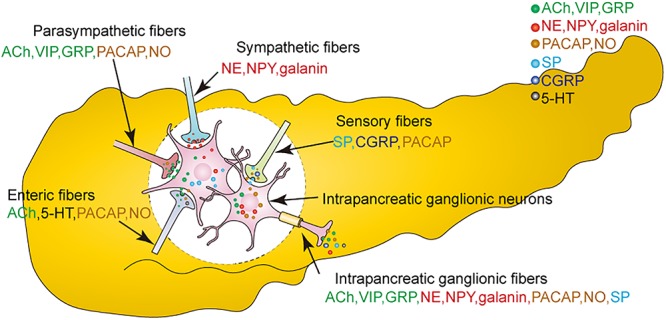
Neurotransmitters are released from both extrinsic nerves and intrinsic intrapancreatic ganglia. Neurotransmitters are, respectively released from parasympathetic fibers, sympathetic fibers, sensory fibers, enteric fibers, the intrapancreatic ganglionic fibers. ACh, acetylcholine; GRP, gastrin-releasing peptide; VIP, vasoactive intestinal peptide; NO, nitric oxide; NE, noradrenaline; NPY, neuropeptide Y; PACAP, pituitary adenylate cyclase activating polypeptide; 5-HT, serotonin; CGRP, calcitonin gene-related peptide; SP, substance P.

A variety of neurotransmitters can be identified in the intrapancreatic ganglia (Figure 2). Studies show that choline acetyltransferase (CHAT)-immunopositive neuronal cell bodies and nerve fibers are present in the pancreas of many species (Liu et al., 1998; Love and Szebeni, 1999a; Wang et al., 1999), suggesting that ACh is not only released from parasympathetic efferents and enteropancreatic fibers, but also from the neurons in the intrapancreatic ganglia. However, intrapancreatic ganglia are not innervated by cholinergic nerve fibers in the sheep pancreas (Arciszewski and Zacharko-Siembida, 2007), which suggests that the cholinergic innervation of islets from pancreatic neurons is species-dependent.

It has been shown that intrinsic noradrenaline-containing neurons are present in the pancreases of rats and newborn guinea pigs. Dense catecholamine-containing nerves with few cell bodies are observed in the intrapancreatic ganglia of rabbits. Moreover, high performance liquid chromatography (HPLC) reveals higher levels of NE in the ganglia of the head and neck regions than in those of the body of the pancreas (Yi and Love, 2005a). It has also been reported that CHAT is colocalized with dopamine-β-hydroxylase (DβH), indicating that ACh and NE can be released from the same neurons. However, the origin of these fibers that release both ACh and NE remains unknown (Liu et al., 1998). Recent studies have also shown an increased density of noradrenergic nerve fibers in the islets of obese mice compared to control mice (Giannulis et al., 2014).

Apart from ACh and NE, many neuropeptides are also released from the intrapancreatic ganglia (De Giorgio et al., 1992a). VIP and GRP are located in both neuronal cell bodies and fibers of the intrapancreatic ganglia (De Giorgio et al., 1992b, 1993; Myojin et al., 2000). VIP is present in most intrapancreatic ganglionic cell bodies, while the number of neuronal cell bodies containing GRP is much lower (De Giorgio et al., 1992b). External PACAP originates from vagal nerves, sensory nerves, or enteropancreatic nerves, while intrinsic PACAP has been observed in the neurons of intrapancreatic ganglia (Hannibal and Fahrenkrug, 2000; Kirchgessner and Liu, 2001). PACAP is co-localized with VIP in the neurons of the rat pancreas (Hannibal and Fahrenkrug, 2000). PACAP is also co-localized with galanin, SP, and corticotrophin-releasing factor (CRF) in the intrapancreatic ganglia of the sheep pancreas (Arciszewski et al., 2015). Moreover, both galanin and NPY can be observed in intrapancreatic ganglia of the bovine pancreas (Furuzawa et al., 1996; Myojin et al., 2000; Adeghate et al., 2001), with galanin being more frequently observed than NPY (Myojin et al., 2000).

Besides sensory nerves, approximately 50% of the SP in the pancreas originates from intrapancreatic ganglia (Shen et al., 2016). CGRP-positive fibers are observed in intrapancreatic ganglia, but not in cell bodies (Silvestre et al., 1996; Myojin et al., 2000). Methionine-enkephalin-positive neurons are occasionally found in bovine intrapancreatic ganglia (Myojin et al., 2000).

Nicotinamide adenine dinucleotide phosphate (NADPH)-diaphorase (d) and nitric oxide synthase (NOS) levels have been used as markers of NO production in the intrapancreatic ganglia (Sha et al., 1995; Liu and Kirchgessner, 1997). This demonstrates that apart from enteric and vagal nerve fibers, nitrinergic nerves are also present in the intrapancreatic ganglia (Wang et al., 1999; Chaudhury, 2014). Most NADPH-d-positive neurons show immunoreactivity for VIP or galanin in the chicken pancreas (Hiramatsu et al., 1994). Moreover, ACh is co-localized with VIP or NOS or both in most cholinergic neurons (Myojin et al., 2000).

## Electrophysiological Properties and Synaptic Potentials of Pancreatic Neurons

The electrophysiological properties of intrapancreatic ganglionic neurons have been characterized through intracellular recording in guinea pigs, rabbits, cats, and dogs (King et al., 1989; Sha et al., 1996; Liu and Kirchgessner, 1997; Sha and Szurszewski, 1999; Love, 2000). The resting membrane potentials (RMP), input resistances (IR), action potential (AP) thresholds, AP amplitudes, time constant, and after-spike hyperpolarization (ASH) durations are similar in different species, as are the electrophysiological properties of pancreatic neurons in different regions of the pancreas (Sha et al., 1996). Electrophysiological properties of pancreatic neurons are summarized in Table 1. Pancreatic neurons have higher IR than other autonomic neurons. Most of the pancreatic neurons are classified as neurons with phasic firing patterns. A small number (9%) of rabbit pancreatic neurons show spontaneous low amplitude oscillations that resemble pacemaker potentials (Love, 2000).

**Table 1:** Electrical properties of pancreatic neurons in different species.

	Guinea pigs Liu and Kirchgessner (1997)	Rabbits Love (2000)	Dogs Sha and Szurszewski (1999)	Cats King et al. (1989)	Region of the cat pancreas Sha et al. (1996)		
					Head	Body	Tail
RMP (mV)	-51.9 ± 0.6	-54 ± 0.4	-53.5 ± 1.6	-49 ± 2	-49.1 ± 7.1	-49.5 ± 7.2	-53.5 ± 1.6
IR (MΩ)	84 ± 2.3	106 ± 6	88.2 ± 1.9	46 ± 4	66.0 ± 31.6	69.1 ± 36.2	60.7 ± 28.4
AP threshold (mV)	-30.5 ± 0.8	-15 ± 1		-33 ± 1			
AP amplitude (mV)	53.2 ± 1.0	65 ± 1		60 ± 1			
Time constant (ms)	1.8 ± 0.1	2.0 ± 0.1		3.1 ± 0.2	3.0 ± 0.1	3.1 ± 0.1	3.1 ± 0.1
ASH amplitude (mV)	12.6 ± 1.1	11 ± 0.5		17 ± 1			
ASH duration (ms)	168.6 ± 23.6	210 ± 19					

*RMP, resting membrane potentials; IR, input resistances; AP, action potential; ASH, after-spike hyperpolarization. All the values represent mean ± standard error of the data*.

Pancreatic neurons exhibit both fast and slow excitatory postsynaptic potentials (fEPSPs and sEPSPs). Stimulation of nerve bundles attached to intrapancreatic ganglia evokes multiple fEPSPs (Sha et al., 1996, 1997; Liu and Kirchgessner, 1997; Love, 2000) that are blocked by hexamethonium in 75% of the neurons, while non-cholinergic fEPSPs are observed in 25% of the neurons (Love, 2000). Repetitive stimulation evokes muscarinic and non-cholinergic sEPSPs, whose duration and amplitude are positively related to the stimulus trains; hexamethonium has no impact on the sEPSPs (Sha et al., 1996; Liu and Kirchgessner, 1997). sEPSPs are evoked by repetitive stimulation using atropine or BRL24924, a 5-HT_1p_ receptor antagonist (Sha et al., 1996, 1997). Spontaneous fEPSPs are usually observed at low frequency, and action potentials (APs) are also observed due to summation of subthreshold fEPSPs (King et al., 1989). Approximately 41% of the intrapancreatic neurons in the head and body of the pancreas show spontaneous fEPSPs and APs, while only 31% of the intrapancreatic neurons in the pancreatic tail show spontaneous fEPSPs and APs (Sha et al., 1996). Pancreatic neurons are capable of coordinating intrinsic activity owing to the rhythmic bursts of fEPSPs and APs in interconnected ganglia (Love, 2000). Inhibitory postsynaptic potentials (IPSPs) have not been reported yet. Intrapancreatic ganglia of rabbits in all three regions of pancreas exhibit paired-pulse facilitation (PPF) or depression (PPD) of fEPSPs. PPF peaking and disappearing have shorter inter-stimulus intervals than PPD. PPF is observed mainly in the pancreatic head and neck (60%), while PPD is observed in the body of the pancreas. In the head/neck region, facilitation during the initial 1–2 s of train stimulation is reduced by mid-train depression (2–5 s) of a greater magnitude. The frequency of postsynaptic APs is inversely related to the magnitude of mid-train depression, suggesting that the electrophysiological activities of pancreatic neurons can regulate synaptic strength. Train stimulation in the head or neck of the pancreas is seen to be followed by brief post-train augmentation of fEPSPs (Sha et al., 1996; Love, 2000; Yi and Love, 2005b). Presynaptic post-train depression results in a decrease in ACh release. Regional differences in electrophysiological activities may reflect regional differences in pancreatic function (Yi and Love, 2005b; Love et al., 2007).

Neurotransmitters of external or internal origin are observed to affect ganglionic transmission. In cats, ACh evokes fast depolarization with a decrease in IR, an effect mediated by nicotinic receptors. Most fEPSPs are activated by nicotinic receptor subunits in ganglionic neurons and few fEPSPs are not sensitive to hexamethonium, indicating that there exist non-cholinergic receptors in the ganglia. The slow transmission is mediated by M_1_ receptors through an increase in the IR (Sha et al., 1997). However, muscarine can convert synaptic APs of rabbit pancreatic neurons into fEPSPs without any changes in the RMP or IR (Love, 2000). The electrical activity of intrapancreatic ganglia has a vital influence on amplitude regulation of pulsatile insulin secretion by isolated ganglia together with the adjacent pancreatic parenchyma (Sha et al., 2001b).

A higher percentage of neurons respond to adrenergic agonists in the pancreatic head than in the body or the tail of the pancreas, a finding consistent with the fact that the density of noradrenergic innervation to intrapancreatic ganglia in the pancreas head is higher than in the body or tail (Yi et al., 2004). A predominance of inhibitory α_2_ receptors in the body of the pancreas contributes to the inhibition of ganglionic transmission, and excitatory α_1_ receptors in the ganglia from the head/neck regions participate in the facilitation of ganglionic transmission. Presynaptic post-train depression (PTD) is mediated by NE and other unknown neurotransmitters (Yi and Love, 2005a).

PACAP depolarizes pancreatic neurons via the activation of ganglionic PAC_1_ receptors and may enhance endocrine secretion (Kirchgessner and Liu, 2001).

While NO evokes membrane hyperpolarization in neurons of the cat pancreas, the membrane IR does not markedly change. Inhibition of NO production may enhance release of ACh to evoke fEPSPs and increase the amplitude of sEPSPs (Sha et al., 1995).

Serotonergic nerve fibers are found in the intrapancreatic ganglia of guinea pigs and cats, but are rare or absent in rabbits (Ma and Szurszewski, 1996b; Liu and Kirchgessner, 1997; Yi et al., 2004). No 5-HT immunoreactive neurons are observed in cat intrapancreatic ganglia (Sha et al., 1996). Neurons in the pancreatic head have a higher excitability in response to 5-HT than those in the tail. The responses of neurons of the intrapancreatic ganglia to 5-HT with fast and slow depolarization are mediated by 5-HT_3_ and 5-HT_1_p receptors, respectively (Ma and Szurszewski, 1996b; Sha et al., 1996).

Cholecystokinin (CCK) has been shown to be present in nerve terminals which surround but are not in the intrapancreatic ganglia. The vagal and sympathetic nerve fibers may be the source of CCK in the pancreas. In cats, cholecystokinin octapeptide (CCK-8) can evoke slow depolarization by acting on CCK_B_ postsynaptic receptors, and amplify nicotinic transmission by facilitating the release of ACh from preganglionic nerve terminals or by increasing the sensitivity of postsynaptic membranes to ACh (Cantor and Rehfeld, 1984; Ma and Szurszewski, 1996a).

Investigation of electrophysiological effects of gamma-aminobutyric acid (GABA) on cat pancreatic neurons indicates that GABA acts on the ganglia through GABA_A_ receptors, which can cause depolarization and inhibit fEPSPs (Sha et al., 2001a). While endogenous GABA has been shown to be stored in and released from the ganglionic glial cells, the origin of the GABA remains unclear (Sha et al., 2001a). Leptin promotes fast synaptic transmission by acting on presynaptic nerve terminals of intrapancreatic ganglia in dogs (Sha and Szurszewski, 1999).

## Neural Regulation of Islets

**Figure 3:**
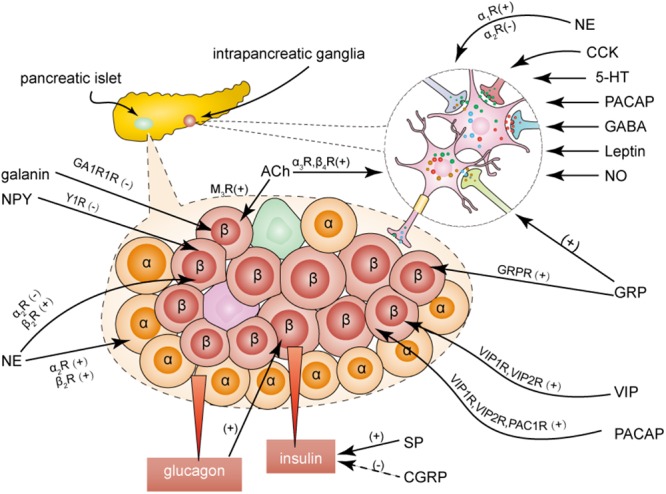
Effects of neurotransmitters on insulin secretion. Neurotransmitters modulate the insulin secretion by activating different receptors both directly at the islets and indirectly at the ganglionic level. ACh, acetylcholine; GRP, gastrin-releasing peptide; VIP, vasoactive intestinal peptide; NO, nitric oxide; NE, noradrenaline; NPY, neuropeptide Y; PACAP, pituitary adenylate cyclase activating polypeptide; 5-HT, serotonin; CCK, cholecystokinin; CGRP, calcitonin gene-related peptide; SP, substance P.

Intrapancreatic ganglia are the integration center for extrinsic nerve inputs and intrinsic neuronal inputs. This integration center plays an important role in regulating pancreatic endocrine function. The extrinsic nerves modulate pancreatic endocrine function either directly at the islet level or by going through the integration center (Gylfe and Tengholm, 2014; Di Cairano et al., 2016; Figure 3).

### Innervation of Islets

The modes of innervation of pancreatic islets vary across species. In mice, the postganglionic parasympathetic fibers contact all types of endocrine cells (α, β, δ, and PP cells) in the islets, while the postganglionic sympathetic fibers innervate α cells and smooth muscle cells of the blood vessels, but not the β cells (Dolensek et al., 2015). The islets are sparsely innervated in adult humans. Parasympathetic postganglionic fibers are observed to contact exocrine tissue preferentially. Most sympathetic nerves innervate smooth muscle cells of the blood vessels in the adult human pancreas. However, the human fetal pancreas is richly innervated (Taborsky, 2011; Krivova et al., 2016). Islet innervation is correlated with age and environmental factors in humans and rats (Proshchina et al., 2014; Mundinger et al., 2016; Tang et al., 2018b). The integration between epithelial cells and components of the nervous system may form the neuro-insular complexes seen during cellular differentiation (Krivova et al., 2016). In addition, the composition and cytoarchitecture of endocrine cells are also different in humans and rats (Rodriguez-Diaz and Caicedo, 2014; Thorens, 2014; Dolensek et al., 2015; Da Silva Xavier, 2018). Current findings indicate that different species may have different mechanisms for neural regulation of pancreatic endocrine function.

### Intrapancreatic Ganglionic Regulation of Islets

As the integrated innervation enters the pancreas, intrapancreatic ganglia directly innervate islets, thereby modulating islet secretion.

Tract-tracing of intrapancreatic ganglionic neurons shows that intrapancreatic ganglia send nerve fibers directly to the islets (Kirchgessner and Pintar, 1991). This ganglion-islet association has been recently demonstrated by a three-dimensional panoramic histology study (Tang et al., 2018b). *In vivo* ganglionic nicotinic activation stimulates insulin and glucagon secretion in the mouse pancreas (Karlsson and Ahrén, 1998). Cholinergic nerves of the intrapancreatic ganglia regulate the oscillatory pattern of basal endocrine secretion (Karlsson and Ahrén, 1998; Fendler et al., 2009). Activation of postganglionic cholinergic nerves by GRP regulates insulin secretion *in vivo* (Karlsson et al., 1998). An electrophysiological study in a ganglion-islet attached preparation setting provides direct evidence for the modulation of insulin secretion by the intrapancreatic ganglia. The activity of intrapancreatic ganglionic neurons directly stimulates insulin secretion, while blockage of this activity removes the modulation (Sha et al., 2001b).

### Sympathetic Regulation of Islets

Sympathetic nerves regulate pancreatic endocrine secretion with multiple direct and indirect mechanisms through their actions on islets, blood vessels, and intrapancreatic ganglia (Yi et al., 2005). Electrical stimulation of sympathetic nerves induces NE release and has an inhibitory effect on basal and glucose-stimulated insulin release by acting on the α_2_-adrenoceptor of β cells in dogs and calves. Sympathetic nerves do not participate in the inhibition of basal insulin secretion in rats, pigs, and humans (Ahrén, 2000). NE can also stimulate insulin secretion by acting on the β_2_-adrenoceptor of β cells or by acting on the α_2_- and β_2_-adrenoceptors of α cells, where glucagon secretion leads to an increase in insulin secretion (Ahrén, 2000). In general, the overall effect of NE is lowering the plasma insulin concentration (Rodriguez-Diaz and Caicedo, 2013). In humans, the primary targets of the sympathetic fibers are vascular smooth muscle cells, which can reduce blood flow to regulate secretion (Rodriguez-Diaz and Caicedo, 2013; Dolensek et al., 2015). Patients with type 1 diabetes show a severe loss of islet sympathetic nerves. Moreover, rats with short-term hyperglycemia show a dysfunction in the islet sympathetic innervation from the celiac ganglia, while hyperphagic weaning mice show markedly increased sympathetic innervation compared to control mice (Mundinger et al., 2015, 2016; Tang et al., 2018b).

Activation of the sympathetic pathway potentiates glucagon secretion and inhibits somatostatin secretion. However, the effects of sympathetic nerve activity on pancreatic polypeptide (PP) secretion are different across species, in that it enhances PP secretion in pig and human pancreases, but inhibits PP secretion in the dog pancreas (Ahrén, 1999; Gilon and Henquin, 2001; Rodriguez-Diaz and Caicedo, 2014).

Both NPY and galanin are known to mediate insulin secretion (Chandra and Liddle, 2013). NPY inhibits glucose-induced insulin secretion in rats and mice, but has no effect in dogs (Dunning et al., 1987; Pettersson et al., 1987; Morgan et al., 1998). Galanin inhibits insulin secretion in dogs but not in humans, as the innervation of galanin-containing nerves is abundant in dog islets, but rare in human pancreatic islets (Ahrén and Lindskog, 1992; Ahrén, 2000). Interestingly, both NPY and galanin have an insulinotropic effect on the pig pancreas (Sheikh et al., 1988). NPY binds to the Y1 receptor subtype, while galanin interacts with GA1R1 receptors in β cells to mediate insulin secretion (Parker et al., 1995; Morgan et al., 1998; Chandra and Liddle, 2009). Oral administration of galanin has been shown to improve insulin sensitivity by decreasing duodenal contraction in diabetic mice (Abot et al., 2018b).

### Parasympathetic Regulation of Islets

Insulin secretion is stimulated by the electrical activation of parasympathetic fibers and inhibited by vagotomy (Ahrén, 2000; Teff, 2008; Balbo et al., 2016). The density of parasympathetic axons is lower in the exocrine tissue of the pancreas in individuals with new-onset type 1 diabetes (Lundberg et al., 2017) than in control individuals. ACh released by postganglionic nerve fibers acts on muscarinic receptors of β-cells and stimulates insulin secretion directly (Van der Zee et al., 1992). The M_3_ receptor subtype plays an important role in regulating cholinergic nerves to induce secretion of islet hormones (Karlsson and Ahrén, 1993; Gautam et al., 2006; Miranda et al., 2016). ACh stimulates glucagon and PP secretion that can be blocked by atropine, and the effect of ACh on somatostatin secretion varies across species (Gilon and Henquin, 2001).

VIP, PACAP, and GRP can stimulate the secretion of glucagon and insulin. Given their similar structures, VIP and PACAP have common receptors called VIP_1_ and VIP_2_ receptors, whereas the PAC_1_ receptor is specific for PACAP (Kirchgessner and Liu, 2001). GRP directly acts on the islets via the GRP receptors (Karlsson and Ahrén, 1998). As the nerves containing VIP are found very close to α cells in dogs, α cells are more sensitive to VIP than β cells (Havel et al., 1997).

### Sensory Nerve-Mediated Regulation of Islets

Sensory nerves regulate endocrine secretion through two mechanisms: (a) direct action on the islet receptors, and (b) formation of neural circuits with adrenergic nerves (Ahrén, 2000). CGRP inhibits glucose-induced insulin release and stimulates glucagon secretion (Ahrén et al., 1987; Silvestre et al., 1996), but the specific CGRP receptor that mediates these processes has not yet been identified. There is considerable variation in reports of the effects of SP on insulin release (Hermansen, 1980; Chiba et al., 1985; Shen et al., 2016). SP induces a marked increase in insulin secretion from the pancreas of healthy rats, but inhibits insulin secretion from the pancreas of diabetic rats, indicating that it may play a role in the onset of diabetes (Adeghate et al., 2001; Shen et al., 2016). It is generally considered that SP interacts with neurokinin G-protein-coupled receptors (NK-R) on islets. In rats, it is suggested that the role of vagal afferents is to suppress insulin secretion (Meyers et al., 2016). The role of sensory nerves in mediating glucose homeostasis remains unexplored.

## Conclusion and Perspectives

Intrapancreatic ganglia constitute endogenous neural networks and release various neurotransmitters. They integrate both intrinsic and external nerve inputs to play an important role in pancreatic endocrine secretion. The mechanisms underlying the role of intrapancreatic ganglia in islet function remain relatively unclear. Further studies are needed to elucidate the complete mechanisms underlying the functions of intrapancreatic ganglia in normal physiological and disease states of the pancreas. This may provide us with novel ways to treat diseases of the pancreas.

## Author Contributions

WL researched the articles and wrote the manuscript. GY, YL, and LS revised the draft before submission. All authors have made direct and intellectual contribution to the work, and approved it for publication.

## Conflict of Interest Statement

The authors declare that the research was conducted in the absence of any commercial or financial relationships that could be construed as a potential conflict of interest.
